# The behavioral response of dairy cows to different shock energies delivered during virtual fencing training

**DOI:** 10.3168/jdsc.2026-1025

**Published:** 2026-04-20

**Authors:** Megan Verdon

**Affiliations:** Tasmanian Institute of Agriculture, College of Sciences and Engineering, University of Tasmania, Tasmania, Australia, 7320

## Abstract

The uptake of virtual fencing (VF) technologies to manage grazing dairy cattle is increasing, but little empirical evidence exists to support acceptable thresholds for the electric shock delivered by these systems. This study quantified the behavioral responses of dairy cows to 4 shock energy levels delivered by the Halter VF system. Forty early-lactation multiparous dairy cows, naïve to VF, were studied in 5 groups of 8 animals. Within each group, cows were randomly allocated to receive one of 4 shock energies (0.1, 0.2, 0.3, or 0.45 J) via a collar-mounted VF unit. Behavioral responses to shock were recorded and classified using a 6-point reaction score: (1) stop, pause, or no response with no steps taken; (2) fewer than 3 steps taken away from the VF; (3) more than 3 steps taken at a normal walking pace; (4) more than 3 steps taken at a fast walking pace and or head shake; (5) move away at a trotting pace; and (6) jump, vocalization, or 360-degree turn. Linear mixed models were used to assess the effects of shock energy, group, and individual cow on behavioral responses. Most reactions (84%) were classified as scores 1 to 3. Shock energy was not associated with reaction score, the number of shocks received, or the time taken to resume grazing within the 0.1 to 0.45 J range studied. Individual cow accounted for 46% of the variation in behavioral response and 34% of the variation in latency to resume grazing. Individual cow characteristics were more influential than shock energy in determining behavioral responses to shock, reinforcing the importance of delivering the lowest effective stimulus for an individual cow in VF systems, without exceeding tested energy thresholds.

## Highlights

• Dairy cow responses to 0.1 to 0.45 J shocks were assessed during virtual fencing.
• Shock energy did not affect intensity of behavioral response.
• Low-intensity responses (scores 1-3) accounted for 84% of observations.
• Individual cow explained 46% of behavioral response.
• Results support use of the lowest effective shock energy in virtual fencing.

## Graphical Abstract

Virtual fencing (VF) technologies manage grazing cattle using an audio cue as the primary signal, with an electric shock delivered if the animal does not respond. Shock characteristics vary across the available VF technologies. There is no evidence describing cow response to different shock energies. In the Halter VF system examined here, training begins at a low shock energy of 0.1 J. Although most cows continue to be managed using low shock energies after training, a small proportion are managed using higher settings, up to 0.45 J. This study examined behavioral responses of dairy cows exposed to 4 shock energy levels (0.1, 0.2, 0.3, and 0.45 J) delivered via Halter VF system. Forty early-lactation dairy cows were observed during their first exposure to the technology. Increasing shock energy did not increase the intensity of aversive behavior, number of shocks received, or time taken to resume grazing. Individual cows differed consistently in responsiveness, supporting use of the lowest effective stimulus.

**Figure:**
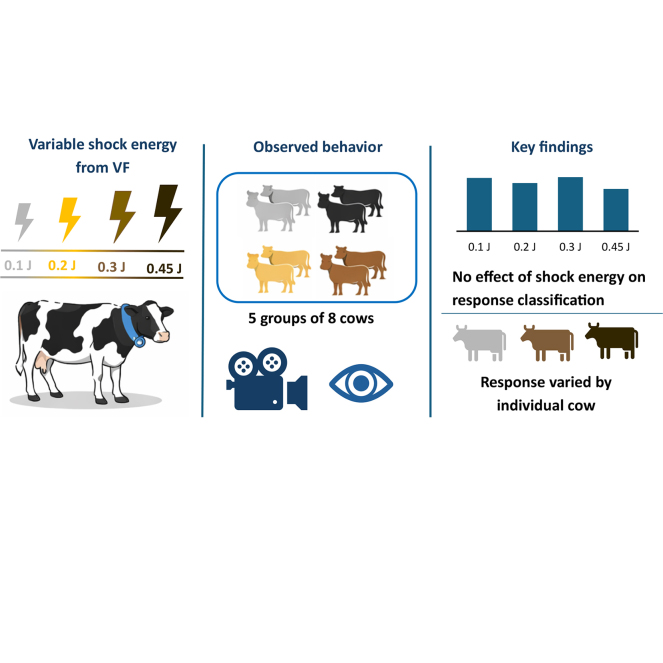

Virtual fencing (**VF**) technologies are increasingly used in grazing dairy systems to manage livestock without physical fences (Wilms et al., 2024). Four commercial VF systems are frequently represented in the scientific literature: Halter (Halter Ltd., Auckland, New Zealand; e.g., Verdon et al., 2024), NoFence (NoFence AS, Batnfjordsøra, Norway; e.g., Harland et al., 2025a,b), eShepherd (Gallagher Animal Management, Hamilton, New Zealand; e.g., Campbell et al., 2019a,b), and Vence (Vence Corp., San Diego, CA; e.g., Antaya et al., 2025). All systems use GPS to determine animal position, accelerometers to monitor movement, and an audio signal as the primary cue, followed by an electric shock if the audio cue is ignored. However, the energy level and duration of electric shock delivery differ across systems. This variation highlights the need to define acceptable thresholds for the electric shock delivered by VF systems.

Rather than deliver a fixed shock energy to all cows, the Halter VF system customizes energy at the individual cow level. Cows are trained using a low shock energy of 0.1 J, which is increased after training is complete only for cows that fail to respond to the cues or later that become nonresponsive. Previous research using Halter technology with an upper shock energy of 0.18 J reported no differences in milk cortisol concentrations or behavior between cows managed with VF and those managed with conventional electric fencing (Verdon et al., 2026). The technology and its commercial application have evolved since that study was conducted in 2023. Current use indicates that 80% to 98% of cows at pasture are managed using a shock energy of less than 0.2 J, depending on the time of year, but a small proportion require shock energies of up to 0.45 J for effective management (S. Shannon, Halter, personal communication, March 19, 2026). This upper threshold has not been evaluated in terms of aversion or associative learning.

The objective of this study was to describe the behavioral indicators of aversiveness of electric shocks delivered by Halter VF technology within the range of 0.1 to 0.45 J. It was hypothesized that the intensity of behavioral responses to shock would increase with increasing shock energy.

To test this hypothesis, an experiment was conducted over 3 d in spring 2025 (October 29–31) at a commercial dairy farm in the northwest of Tasmania, Australia. Forty early-lactation multiparous dairy cows were studied in 5 groups of 8 animals each. Cows were selected from the larger herd of ~500 cows on the basis that they were over 30 DIM and had not been treated for lameness, mastitis, or metritis in the preceding fortnight. Farm records did not enable selection of cows based on age or milk production, but the farm manager used their local knowledge to ensure variability in these characteristics.

The experiment was conducted independently by the primary researcher. The commercial study farm was identified through Halter, as their planned training timeline aligned with the proposed research. However, the farm itself was independently approached and recruited by the researcher. Study design, data analysis, and interpretation were determined solely by the researcher. Halter had no role in experimental design, statistical analysis, or interpretation of the results. Halter's involvement was limited to the deployment of collars, configuration of shock energy settings, and provision of device records following completion of the study, including timestamps of audio cue and electric shock delivery. The researcher retained full control of the dataset and analytical workflow. The researcher remained blinded to shock energy allocation during behavioral observations and statistical analyses, with treatment identities revealed only after all analyses were finalized. Halter reviewed the manuscript for technical accuracy and protection of proprietary information.

All cows were naïve to the VF technology when recruited and were fitted with Halter collars (P5 model) the day before the start of this experiment. Four shock energy levels (0.1 J, 0.2 J, 0.3 J, 0.45 J) were assigned to collars, so that 2 cows were randomly allocated per energy level per group of 8 cows, giving a total of 10 cows per level across the experiment. According to third party bench testing, the shock energy delivered by the Halter technology is within ±10% of the programmed energy (R. Power, Halter, personal communication). In situ verification of individual collar output was not performed in the present study.

A prior power analysis calculation was conducted using baseline behavioral data reported by Hamidi et al. (2024) at 0.2 J shock energy. In that study, cows were assigned a reaction score on a 4-point scale based on their behavioral response to the shock delivered through VF, where 1 indicated effective containment with minimal or no behavioral response and 4 indicated ineffective containment with a pronounced behavioral response. Power analysis based on the observed variability reported in Hamidi et al. (2024) and assuming α = 0.05 and power = 0.80, the present design (n = 10 cows per energy level) was sufficient to detect a biologically relevant moderate difference between treatments (≥0.7 shift in average behavior score).

Two adjacent paddocks separated by a shared laneway were used for this study. Each paddock was enclosed by permanent 2-wire electrified fencing. The front 40 m of both paddocks was segregated from the rest of the paddock using temporary electric fences, forming a sub-paddock. These were again divided using electric back-fences and virtual front-fences to create grazing cells for the behavioral observations. Only one group grazed a cell at any given time.

Groups alternated between paddocks such that on day 1 of testing, group 1 grazed in paddock 1, group 2 in paddock 2, and group 3 in paddock 1. On day 2, group 4 grazed in paddock 1, and group 5 grazed in paddock 2. Each grazing cell contained a water trough and provided an over-allocation of pasture. The location of the electric and virtual fences changed between groups to provide a new grazing cell with fresh grass for each test. The virtual fence was always activated over a fresh allocation to promote grazing and interaction with the technology.

Selected cows were drafted from the main milking herd on the day before testing, restrained in a crush, marked on their shoulders with stock spray for individual identification, and fitted with a Halter collar. The collars remained inactive at this stage. The 40 cows were then moved to a temporary paddock overnight in preparation for testing the following day.

On day 1 of testing, the 40 cows were held on a feed-pad with ad libitum silage after milking. At 0900 h, all cows were moved from the feed-pad to a nearby paddock. Eight cows were drafted from the group of 40 and moved to the first grazing cell in paddock 1. The cows were held within the cell using temporary electric back and front fences for 10 min to allow them to settle. During this period, the collars were activated, the energy treatments were randomly assigned to collars, and the virtual fence was established. Once the collars had synchronized (i.e., received the information about the assigned energy level and the virtual fence location), the temporary electric front fence was removed and replaced with a virtual fence, while retaining the electric back fence (this occurred at 0915 h). The electric front fence was then re-established 10 m beyond the virtual fence location, providing cows with a visual cue to the virtual fence position, consistent with Halter's standard training procedures (e.g., Verdon et al., 2024, 2026).

Cows were left in the paddock to freely explore, graze, and interact with the virtual fence. After one hour, the boundary positions of the virtual and electric fences were reversed. That is, the virtual fence was moved to the location of the electric back fence, and the electric back fence was moved to the previous location of the virtual fence. This re-stimulated grazing behavior and increased the likelihood that cows would interact with the virtual fence while maintaining grazing within a single observation area, so that all interactions were visible to the researcher. Cows were then observed for an additional hour.

Collars were deactivated at the end of the 2-h observation period, and cows were returned to the main milking herd. Another 8 cows were then randomly drafted from the remaining 32 animals, moved into the grazing cell in paddock 2, and observed following the same procedures (group 2 start time: 1130 h). When group 2 finished, a third group of 8 animals was tested in paddock 1 (group 3 start time: 1350 h). All testing was concluded before the afternoon milking finished, and cows were returned to the main herd after testing.

The same procedures were followed on day 2 using the remaining 16 naïve cows (groups 4 and 5). Group 4 was tested in paddock 1 (start time: 0915 h) and group 5 in paddock 2 (start time: 1130 h). After testing, all cows re-joined the commercial milking herd and resumed normal daily routines.

The behavioral responses of individual cows to shock delivered by the VF collars were continuously observed in situ by the researcher, who has over 8 years of experience observing cattle behavior in VF systems. The researcher was blind to treatment. Responses to the electric shock were recorded by the researcher using a predefined ethogram describing locomotor and behavioral reactions occurring within 5 s of shock delivery. Behaviors included pausing or stopping, taking fewer or more than 3 steps, walking away, fast walking, trotting, head shaking, jumping, vocalization, and rapid turning movements (i.e., 360-degree spin).

Direct observations were supplemented by video footage collected using GoPro action cameras (GoPro Hero 10 Black action camera, GoPro Inc., San Mateo, CA). For both live and video observations, an interaction resulting in electric shock was defined as an audio cue followed by an involuntary muscle twitch, usually with a clear change in behavior. Both the audio cue and the shock were audible during the live observations, although these sounds were not consistently captured on video.

After the study concluded, Halter records detailing the time, date, and type of stimulus delivered (audio or shock) to each cow were supplied and used to supplement the observational data. Halter records did not specify the cows' energy treatment group at this point. The Halter records, together with the video footage, were used by the researcher to verify the direct observations, identify any interactions that were missed in situ, and determine the time taken for each cow to resume grazing after receiving a shock. The latter was taken as the time in seconds from when the cow flinches from the shock to the resumption of grazing behavior. Cows that continued grazing after receiving a shock were given a latency of 0 s in time to resume grazing. Latency to resume grazing was not recorded in instances where cows were interrupted by, for example, another cow in the group or distraction by a nearby mob.

The behavioral response of each cow to each shock was then classified using a 6-point scale: (1) stop, pause, or no response, no steps; (2) <3 steps; (3) >3 steps, normal pace; (4) >3 steps, fast walk, head shake; (5) >3 steps, trotting pace; (6) jump, vocalization, turn 360 degrees. Behavioral classifications 1 to 3 are considered low-intensity responses as typically observed in a VF system. Classifications 4 to 6 represent higher-intensity responses that should be minimized and ideally eliminated. This classification system was derived from the existing literature (Verdon et al., 2021a; Hamidi et al., 2024) and observer experience. When a cow displayed behaviors spanning multiple categories, the highest relevant classification was recorded. Hereafter, behavioral classifications will be referred to as the reaction score.

The researcher remained blind to the treatment during statistical analysis. Halter identified which cows were allocated to the same treatment group but did not reveal which energy level corresponded to these treatment groups until after the analysis was finalized. All analyses were conducted using linear mixed models in SPSS (IBM SPSS Statistics 29.0.0.0). The statistical significance level was set at α ≤ 0.05.

Distributions of reaction scores within and between groups are presented descriptively. A chi-squared test of independence was used to examine the distribution of reaction scores over shock energy levels. Reaction scores 5 and 6 occurred infrequently, so were collapsed into a single category for this analysis to ensure adequate expected cell counts for analysis.

Linear mixed models were used to assess the effects of shock energy on cow behavior and the number of shocks received. Reaction scores and latency to graze were averaged over all the shocks received for each cow, producing one data point per measure per animal. The log_10_-transformed (number of shocks), log_10_(average reaction score), and log_10_(average time to resume grazing) were used as dependent variables, in separate models. Each model included the fixed effects of shock energy (0.1 J, 0.2 J, 0.3 J, 0.45 J), group (1–5), and their interaction. The interaction term was nonsignificant in all models and was removed, with final models including main effects only.

Repeatability of reaction score and latency to graze across consecutive shocks were analyzed using mixed models. The dataset was restricted to the first 4 shocks received, which captured 89.7% of cows. The first model used log_10_(reaction score) as the dependent variable, and the second used log_10_(time to resume grazing plus one). Both models included the fixed effects of shock number (1–4), shock energy (0.1–0.45 J), and group (1–5). The shock number × energy interaction was nonsignificant and removed from all models. Cow was included as a random effect with repeated observations over shock number accounted for using an autoregressive covariance structure. The proportion of total variance in each model attributable to individual cows was quantified using the intraclass correlation coefficient, calculated as the ratio of cow-level variance to total variance.

Each model fit was evaluated and deemed suitable using Akaike information criterion values and by examining the distribution of residuals and residual-versus-predicted scatter plots. The results of these analyses are outlined below, after first presenting details regarding data completeness.

The camera failed during the first hour of observations for group 2 and for 5 min in group 4. Twenty-five of the 51 shocks in group 2 and 11 of the 55 shocks in group 4 occurred during these periods. Behavioral responses were still recorded through direct observation, but the time to graze could not be obtained for these shocks. A further 36 time to graze data points in total were missing due to poor visibility of the focal cow (for example, blocked by other cows), distraction caused by nearby mobs, or social interactions that interrupted normal grazing. In total, 18.8% of time to graze measurements could not be captured. One cow in group 1 (0.2 J energy treatment) did not interact with the virtual fence, and thus no data were recorded for this animal.

Over the course of the observation periods, a total of 285 shocks were delivered across the 5 groups of cows, ranging from 32 in group 1 to 80 in group 5. Excluding the single cow that did not interact with the virtual fence, cows received an average of 7.4 shocks each over the 2-h observation period (range 3–16 shocks).

Nearly half of all recorded responses to these shocks involved cows walking more than 3 steps away from the fence at a normal pace (reaction score 3; 46.7%), and a further 27.4% involved turning away and taking 3 or fewer steps (reaction score 2). The lowest ranked reaction score of 1 (no steps taken) accounted for 9.8% of all responses, taking the total proportion of typical or low-intensity responses observed to 83.9%.

Of the more reactive responses recorded, a reaction score of 4 (fast walk or head shake) accounted for 11.2% of total observations. Eight responses (3.2%) were classified as a reaction score of 5, indicating movement faster than a walk. Seven of these were from 2 cows in the same group (group 5) that consistently showed signs of heightened sensitivity to shock. The only 2 “jump” responses and all 4 of the level 6 reaction scores were also from these cows, after which one collar was paused (cow 2062). These high-intensity responses (reaction scores 5–6) were observed in cows assigned the 0.3 J (cow 2062, group 5), 0.1 J (cow 3734, group 5), and the 0.45 J treatments (cow 383, group 3). No vocalizations were recorded.

The distribution of reaction scores differed over the 4 shock energy levels (Figure 1, χ^2^ = 29.3, df = 12, *P* = 0.004), with a small-to-moderate effect size (Cramer's V was 0.185). Reactions scores of 5 and 6 were disproportionately more common at energy 0.3 J, all due to the responses of cow 2062, whose collar was paused. This relationship was not significant when cow 2062 was removed from the dataset (χ^2^ = 18.6, df = 12, *P* = 0.07).

**Figure 1:**
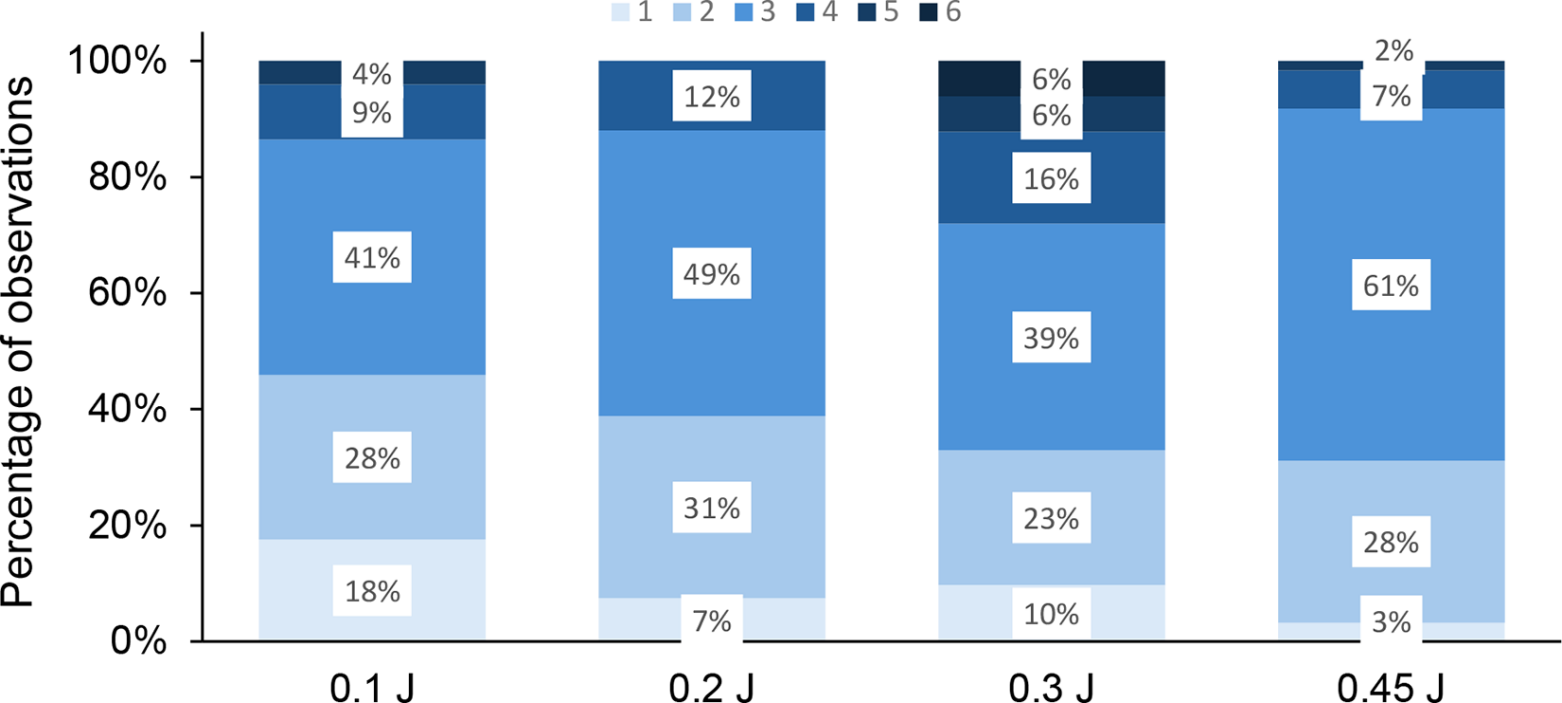
Distribution of reaction scores 1 to 6, represented by the different shades of blue, at each of the 4 energy levels. Reaction scores can briefly be summarized as (1) stop, pause, or no response, no steps; (2) <3 steps; (3) >3 steps, normal pace; (4) >3 steps, fast walk, head shake; (5) >3 steps, trotting pace; and (6) jump, vocalization, turn 360 degrees.

There was no significant effect of shock energy on the number of shocks received, the average reaction score, or the average time taken to resume grazing (Table 1). Group differences were evident only for the number of shocks (F_4,31_ = 3.13, *P* = 0.028), with cows in group 5 receiving approximately twice as many shocks as those in group 1 (9.8 vs. 4.5 shocks, back-transformed means). The models for the number of shocks received and the time to resume grazing explained 33% and 24% of the total data variance, respectively. The model for the average reaction score explained only 9% of the variance.

**Table 1:** Model-estimated means and 95% confidence limits (back-transformed) for number of shocks, average reaction scores, and average time to resume grazing by shock energy treatment; raw means are presented in italics

Variable	Energy level (J)				*F* (df)	*P*-value
	0.1	0.2	0.3	0.45		
Number of shocks	6.6 (5.0–8.8)	6.7 (4.9–9.0)	7.5 (5.6–9.9)	5.5 (4.2–7.3)	*F*_3,31_ = 0.8	0.49
	*6.0*	*6.0*	*5.6*	*5.3*		
Reaction score	2.6 (2.2–3.0)	2.6 (2.2–3.0)	2.9 (2.5–3.4)	2.8 (2.4–3.3)	*F*_3,31_ = 0.6	0.60
	*2.5*	*2.5*	*2.8*	*2.7*		
Time to graze (s)	8.3 (5.8–11.9)	14.2 (9.7–20.8)	13.0 (9.0–18.6)	11.3 (7.9–16.2)	*F*_3,31_ = 1.7	0.18
	*8.5*	*13.6*	*12.4*	*10.9*		

Reaction scores were not associated with energy treatments (*F*_3,31_ = 0.57, *P* = 0.64) or successive shocks (*F*_3,95_ = 2.08, *P* = 0.11; Figure 2A). The random intercept for cow accounted for 46% of the total variance in reaction score, indicating moderate individual consistency in responsiveness. Group effects were negligible (*F*_4,31_ = 0.45, *P* = 0.77).

**Figure 2:**
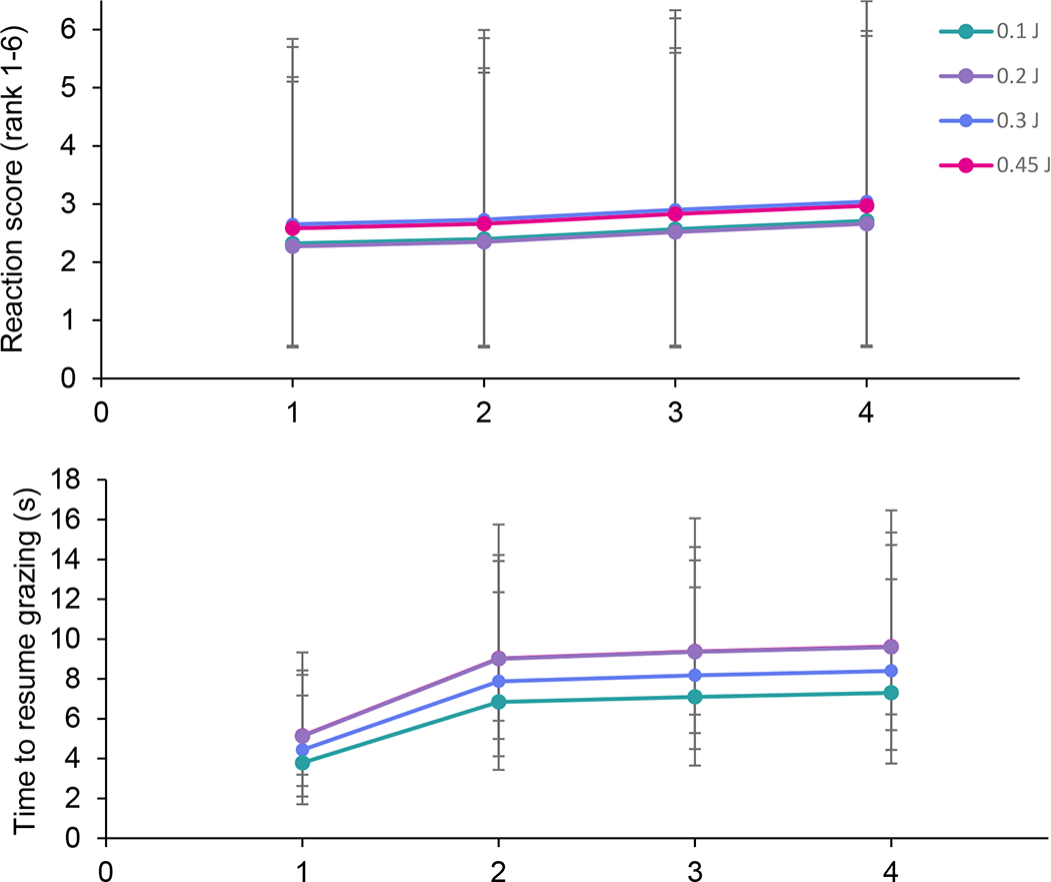
Back-transformed model-estimated means (±95% CI) for reaction score and time to resume grazing following the first 4 successive shocks (x-axis) across energy treatments.

Shock number significantly affected the time taken to resume grazing (*F*_3,70.5_ = 3.59, *P* = 0.018; Figure 2B). Compared with the first shock, latency to graze was 1.63×, 1.69×, and 1.73× longer following the second, third, and fourth shocks, respectively, although these latter shocks did not differ from each other (Figure 2B). Neither energy treatment (*F*_3,28.3_ = 0.29, *P* = 0.83) nor group (*F*_4,29.4_ = 0.74, *P* = 0.57) had significant effects. The random intercept for cow in this model explained 34% of total variance, suggesting that approximately one-third of variation in time to resume grazing reflected between-cow differences.

Time to resume grazing increased with reaction scores 1 to 3 (average of 3.1, 6.9 and 15.6 s for reaction scores 1, 2 and 3 respectively), but there was no further increase with scores 4, 5, and 6 (average of 21.0, 14.4, and 12.5 s for scores 4, 5, and 6, respectively). This is consistent with the locomotory progression from zero steps, to fewer than 3 steps, to a movement of more than 3 steps.

Taken together, the results presented above indicate that, under the experimental conditions used, a shock energy between 0.1 and 0.45 J delivered through the Halter VF collar was not associated with differences in cow behavioral response to shock, the number of shocks received, or the time taken to resume grazing after receiving a shock. These observations are consistent with established principles of aversion learning, that once an aversive stimulus is sufficiently intense to be clearly perceived, further increases in intensity produce diminishing changes in avoidance behavior (Bitterman, 1975). To be clear, these findings should not be interpreted as demonstrating equivalence among energy levels or defining an upper threshold beyond the specific range examined here. Rather, they indicate that a detectable threshold for increased behavioral reactivity was not observed within the range of 0.1 to 0.45 J evaluated in this study.

Nearly half the variation in reaction score and one-third in latency to resume grazing were due to the individual cow. Variability in how cattle interact with and respond to cues delivered by VF systems is well documented (e.g., Campbell et al., 2019a,b; Verdon et al., 2021a,b, 2024). The source of this variation is multifactorial and reflects differences in how individual animals perceive the electric stimulus. Factors shown to influence response to shock include temperament (Verdon et al., 2020), previous experience with electric fencing (Verdon et al., 2020), age (Verdon and Rawnsley, 2020), hunger (Colusso et al., 2021), and social motivation (Colusso et al., 2020; Verdon et al., 2021b). The between-cow variability observed in the present study, consistent with the previous reports of individual differences in cow responses, supports the need for VF systems to deliver the lowest effective stimulus at the individual animal level while remaining within experimentally tested energy ranges. Importantly, the present short duration study does not allow inference about longer term learning outcomes or the welfare implications of individual differences in interaction with the virtual fence, which remain important areas for further research.

Although the shock energy level was not tailored to individual cows in this study, 84% of responses were ranked as low-intensity (score 1–3). This aligns with Hamidi et al. (2024), who observed that 82.4% of 0.2 J shock responses involved cows moving fewer than 3 steps or that continue grazing. The proportion of stronger behavioral responses was also similar between studies, with 3.2% trot responses in the present work and 4.1% in Hamidi et al. (2024). These figures represent an improvement on earlier VF prototypes where 27% of responses involved trotting (Verdon et al., 2021a; 0.2 J). The present study captured cows very early during their first exposure to the VF system. Hamidi et al. (2024) and Verdon et al. (2021a) observed increasing mild behavioral responses and decreasing strong responses as cows gained experience with the system. Accordingly, the distribution of responses seen in this study reflects behavioral reactions during very early exposure, when responses are typically most pronounced.

During the observation period, one cow displayed several 360-degree turning movements after receiving a shock, and consequently her collar was paused. This behavior is consistent with confusion, but may also be an artifact of experimental design. In the present experiment, cows were removed from their normal herd environment and trained in small groups within a confined area with frequent fence shifts. These conditions were necessary to ensure repeated interactions with the virtual boundary so that behavioral response to shock could be recorded; however, they also produced a higher rate of interactions than typically observed under commercial conditions. For example, previous research reports that cows generally receive from 2 to 10 shocks during the first 24 h of training (e.g., Lomax et al., 2019; Langworthy et al., 2021; Hamidi et al., 2022; Verdon et al., 2024; Fuchs et al., 2025), compared with an average of 7.5 shocks/cow over the 2-h observation period in this study. Although the 360-degree turnings response may have been influenced by testing conditions, it highlights the critical importance of safeguards that detect atypical behaviors and pause cue delivery, allowing for the animal to be assessed.

Some social instability was observed in the groups studied. For example, the cow in group 5 whose collar was paused appeared reluctant to rejoin her mob and was seen being displaced and receiving aggression both before and after the collar was paused. Group composition in this study was determined by convenience (the first 8 cows drafted were tested) rather than random assignment, which may have resulted in more anxious or avoidant individuals remaining in the later groups. This could partly account for the higher number of shocks in group 5 compared with group 1. Nevertheless, energy treatments were assigned within groups, and group was not a significant predictor of reaction score or time to resume grazing in any of the statistical models.

The longer latencies to resume grazing following the first shock likely reflect short-term vigilance or attentional shifts rather than increasing reactivity. Latency to resume grazing has been used as an indicator of shock aversiveness (e.g., Quigley et al., 1990; Hamidi et al., 2022). However, the present results suggest it is a noisy proxy for cow emotional state following a shock. Latency increased with the amount of locomotion the cow performed, but not with the valence of the behavioral response (e.g., jumps, trot, 360-degree spin). It was also affected by grazing motivation, social interactions, brief distractions, and pasture distribution. Thus, latency to resume grazing may reflect a short-lived pause for caution or situational assessment rather than a direct measure of how negative the shock was perceived by the cow. Where possible, pairing latency with complementary indicators, such as changes in heart rate and heart rate variability, would provide a more robust assessment of valence.

In conclusion, under the experimental conditions of this study, shock energies between 0.1 and 0.45 J delivered through the Halter VF collar were not associated with differences in the intensity of behavioral response, the number of shocks received, or the time to resume grazing.
